# A closed loop audit of clerking psychiatric histories in an acute psychiatric inpatient unit

**DOI:** 10.1192/bjo.2021.300

**Published:** 2021-06-18

**Authors:** Samuel Richard Smith, Rajiv Ark, Thirunavukkarasu Aravinth

**Affiliations:** 1 Lancaster University, Lancashire & South Cumbria NHS Foundation Trust; 2 Lancashire & South Cumbria NHS Foundation Trust, Lancaster University; 3 Lancashire & South Cumbria NHS Foundation Trust

## Abstract

**Aims:**

An accurate and complete history is a key component of a medical consultation. Evidence suggests that up to 80% of diagnosis may be made entirely off the patient history. The aim of this closed loop audit was to examine the effects of a clerking pro forma on the quality of doctors clerking histories of new patients admitted to an acute psychiatric inpatient unit, against standards suggested in the New Oxford Textbook of Psychiatry.

**Method:**

Data for this audit were gathered by finding the initial clerking history for inpatients at The Orchard on ECR and RIO. The clerking histories of the 18 inpatients present on 12.10.20 were initially audited. These standards recommend in the in the New Oxford Textbook of Psychiatry include; Patient Identification (ID), Presenting Complaint (PC), History of Presenting Complaint (HPC), Psychiatric history, Medical history, Family history, Forensic history, Social history, Personal history, Premorbid personality, Mental state exam (MSE). After analysis of the results of the first loop, a clerking pro forma was created and distributed to junior doctors to implement. The clerking histories for the subsequent 18 patients to be admitted were then audited and compared.

**Result:**

The results of the first audit cycle were poor. Only patient identification and presenting complaint were present in 100% of clerked histories. Concerningly, only 72% of the histories included the patients’ medical histories, forensic histories were included 44% of the time, and social history just 39% of the time.

The implementation of a clerking history proforma showed improvements in all areas of clerking. Patient ID, PC, HPC, psychiatric history and MSE were now present in 100% of clerked histories. Forensic history showed a statistically significant improvement from 44% to 73% [X2(1) = 5.9; p = 0.015]. Social history showed a statistically significant improvement from 39% to 78% [X2(1) = 5.6; p = 0.018]. Premorbid personality showed a statistically significant improvement from 44% to 89% [X2(1) = 8.0; p = 0.005]. Personal history showed a non-statistically significant improvement from 39% to 56%, as did medical history from 72% to 94%, and family history from 39% to 61%.

**Conclusion:**

In conclusion, the implementation of a clerking history pro-forma has significantly improved the quality and completeness of clerking histories gathered by doctors at The Orchard. This is hopefully increase diagnostic accuracy and improve the quality of care of patients in the hospital.

